# Preconception interventions and resources for women with serious mental illness: a rapid evidence review

**DOI:** 10.1192/bjo.2021.626

**Published:** 2021-06-18

**Authors:** Katie Atmore, Louise Howard, Abigail Easter

**Affiliations:** IoPPN King's College London

## Abstract

**Aims:**

There is little research into evidence-based preconception interventions for women with serious mental illness (SMI). Women with SMI will have specific needs around preconception due to the complexities of the teratogenicity of medications, risk of mental illness relapse and higher levels of stigma around motherhood. If effectively delivered preconception care could mitigate these difficulties and improve outcomes for mother and baby. The aim of this research was therefore to determine to identify and describe studies evaluating preconception interventions for women of child-bearing age who have an existing SMI through searches of the peer-reviewed literature.

**Method:**

A rapid review was conducted to search MEDLINE and PsychINFO databases from the year 2000 onwards for peer-reviewed articles describing preconception interventions/resources delivered prior to a pregnancy to women of child-bearing age with a pre-existing existing serious mental illness (including schizophrenia, bipolar and eating disorders).

**Result:**

A total of 592 results were returned from the searches and 576 of these remained after the removal of duplicates. 11 studies were included in the final narrative synthesis describing the following intervention types: Health warning (1), Health screening (1), Teratogen phone service (2), Psychiatric consultation (5), Family planning information (1) and Peripartum management plan (1). Interventions were delivered in Australia, UK, Italy, Germany, Netherlands, USA and Nigeria.

**Conclusion:**

Though the included studies indicated that some efforts have been made globally to meet the preconception needs of women with SMI the numbers included in the studies tended to be low and reflective of small-scale service provision. Future studies utilising a randomised controlled trial design would lower the risk of bias and provide more generalisable evidence of effectiveness for these interventions. The results of this review were used to inform the development of a number of resources to aid the planning of healthy pregnancies in both women with SMI and the health professionals working with them.

